# The histone deacetylase inhibitor trichostatin a decreases lymphangiogenesis by inducing apoptosis and cell cycle arrest via p21-dependent pathways

**DOI:** 10.1186/s12885-016-2807-y

**Published:** 2016-09-30

**Authors:** Igor Hrgovic, Monika Doll, Johannes Kleemann, Xiao-Fan Wang, Nadja Zoeller, Andreas Pinter, Stefan Kippenberger, Roland Kaufmann, Markus Meissner

**Affiliations:** 1 Department of Dermatology, Venereology and Allergology, Goethe University, Theodor-Stern Kai 7, Frankfurt/Main, 60590 Germany; 2 Department of Pharmacology & Cancer Biology, Duke University School of Medicine, C218 LSRC, Box 3813, Durham, NC 27710 USA; 3 Klinik für Dermatologie, Venerologie und Allergologie, Klinikum der J. W. Goethe-Universität, Theodor-Stern-Kai 7, Frankfurt am Main, D-60590 Germany

**Keywords:** Histon deacetylase inhibitors (HDACi), Lymphangiogenesis, G0/G1 cell cycle arrest, p21, intrinsic apoptotic pathway

## Abstract

**Background:**

The formation of new lymphatic vessels provides an additional route for tumour cells to metastasize. Therefore, inhibiting lymphangiogenesis represents an interesting target in cancer therapy. First evidence suggests that histone deacetylase inhibitors (HDACi) may mediate part of their antitumor effects by interfering with lymphangiogenesis. However, the underlying mechanisms of HDACi induced anti-lymphangiogenic properties are not fully investigated so far and in part remain unknown.

**Methods:**

Human lymphatic endothelial cells (LEC) were cultured in vitro and treated with or without HDACi. Effects of HDACi on proliferation and cell cycle progress were analysed by BrdU assay and flow cytometry. Apoptosis was measured by quantifying mono- and oligonucleosomes in the cytoplasmic fraction of cell lysates. In vitro lymphangiogenesis was investigated using the Matrigel short term lymphangiogenesis assay. The effects of TSA on cell cycle regulatory proteins and apoptosis-related proteins were examined by western blotting, immunofluorescence staining and semi-quantitative RT-PCR. Protein- and mRNA half-life of p21 were analysed by western blotting and quantitative RT-PCR. The activity of the p21 promoter was determined using a dual luciferase assay and DNA-binding activity of Sp1/3 was investigated using EMSA. Furthermore, siRNA assays were performed to analyse the role of p21 and p53 on TSA-mediated anti-lymphangiogenic effects.

**Results:**

We found that HDACi inhibited cell proliferation and that the pan-HDACi TSA induced G0/G1 arrest in LEC. Cell cycle arrest was accompanied by up-regulation of p21, p27 and p53. Additionally, we observed that p21 protein accumulated in cellular nuclei after treatment with TSA. Moreover, we found that p21 mRNA was significantly up-regulated by TSA, while the protein and mRNA half-life remained largely unaffected. The promoter activity of p21 was enhanced by TSA indicating a transcriptional mechanism. Subsequent EMSA analyses showed increased constitutive Sp1/3-dependent DNA binding in response to HDACi. We demonstrated that p53 was not required for TSA induced p21 expression and growth inhibition of LECs. Interestingly, siRNA-mediated p21 depletion almost completely reversed the anti-proliferative effects of TSA in LEC. In addition, TSA induced apoptosis by cytochrome c release contributed to activating caspases-9, −7 and −3 and downregulating the anti-apoptotic proteins cIAP-1 and −2.

**Conclusions:**

In conclusion, we demonstrate that TSA - a pan-HDACi - has distinct anti-lymphangiogenic effects in primary human lymphatic endothelial cells by activating intrinsic apoptotic pathway and cell cycle arrest via p21-dependent pathways.

**Electronic supplementary material:**

The online version of this article (doi:10.1186/s12885-016-2807-y) contains supplementary material, which is available to authorized users.

## Background

Histon deacetylases (HDACs) are highly conserved enzymes involved in the control of acetylation of histones and nonhistone proteins by which mammalian cells regulate transcriptional activity. The epigenetic control of gene expression results from the balance between the opposing activities of histone acetyltransferases (HATs) and HDACs. Histone hyperacetylation is associated with increased transcriptional activity, whereas decreased levels of acetylation are associated with the repression of gene expression. Eighteen human HDACs have been identified so far and are subdivided into four classes. HDAC 1, 2, 3 and 8 belong to class I, HDAC 4, 5, 6, 7, 9 and 10 belong to class II, class III consists of sirtuins (SIRT1-7) and to class IV belongs to HDAC 11 [1, 2]. Increased HDAC activity is associated with the initiation and progression of various types of cancer by dysregulation of genes which are involved in differentiation, cell cycle control, apoptosis and DNA replication. Aberrant expression and activity of HDACs was found in various cancer entities including melanoma, breast cancer, gastric cancer, prostate cancer and acute myeloid leukemia [3, 4]. A dysregulation of the HDAC/HAT-system leads to the induction of tumourangiogenesis. Several natural and synthetic HDACi have been identified so far and have been shown to inhibit the enzymatic activity of HDACs. HDACi exhibits anti-cancer effects by inducing differentiation, growth arrest or apoptosis in a number of tumour cell lines in vitro. HDACi have to been found to inhibit anti-apoptotic genes (e.g. bcl-XL and bcl-2) and induce pro-apototic genes (e.g. Bax, Bim, CD95 and CD95L) in several tumour cell lines including glioblastoma, lymphoma, prostate cancer and non-small-cell lung carcinoma. In addition, HDACi induce genes which are involved in the control of differentiation, proliferation and the cell cycle (e.g. p21^WAF1/KIP1^, p27^KIP1^, p53, cyclin A, B and D) [5]. Furthermore, HDACi have been shown to reduce angiogenesis by modulating angiogenesis-related genes, such as hypoxia inducible factor-1α (HIF-1α), vascular endothelial growth factor (VEGF) and endothelial nitric oxide synthase (eNOS) [6]. So far, four compounds exhibiting activity against HDACs – vorinostat (suberoylanilide hydroxamic acid; SAHA), romidepsin (FK228; depsipeptide), belinostat (PXD-101) and panobinostat (LBH-589) – have been approved by the Food and Drug Administration for the treatment of relapsed cutaneous T-cell lymphoma, peripheral T-cell lymphoma and multiple myeloma [7]. Several clinical trials of HDACi are underway [8]. Lymphangiogenesis, like angiogenesis, is a complex process of formation of new lymphatic vessels from pre-existing lymphatic vessels. In the adult organism, lymphangiogenesis is under physiological conditions restricted to the endometrium during pregnancy, wound healing or regeneration, tissue repair and inflammation [9]. Recent studies have identified lymphangiogenesis as an important pathophysiological factor of tumour progression and prognosis in cancer patients. It has been found that increased tumoural lymphatic vessel density is associated with poor prognosis, a disease free condition and overall survival in patients with melanoma, breast cancer and colorectal cancer [10]. Therefore, inhibiting lymphangiogenesis represents an interesting target in cancer therapy. First evidence in an artificial immortalized lymphatic endothelial cell model has demonstrated anti-lymphangiogenic activities of HDACi [11]. Taking into account that HDACi are promising compounds with anti-tumorigenic activities, we addressed this issue and tried to elucidate the underlying molecular mechanisms of anti-lymphangiogenic properties by HDACi in primary human lymphatic endothelial cells, as closest model to in vitro conditions.

## Methods

### Cell culture

Human dermal lymphatic endothelial cells were purchased from AngioBio (Del Mar, CA, USA). The cells were isolated from the dermis of juvenile foreskin from different donors. The cells were cultured until the eight passage at 37°C and 5% CO_2_ in an endothelial cell growth medium supplemented with 10% human serum (Lonza, Basel, Switzerland). All experiments have been replicated in different cells from different donors.

### Ethics statement

Primary human material was not used in this study. All work presented has been performed in established, commercially available primary cells.

### Chemicals

Recombinant VEGF-A and VEGF-C were purchased from ReliaTech (Wolfenbüttel, Germany). Sodium Butyrate (NaB), Trichostatin A (TSA) and Valproic Acid (VPA) were obtained from Merck Millipore (Billerica, MA, USA).

### Fluorescence-activated cell sorting analysis

Cells were incubated with 400 nM TSA and Ethanol as a control for 24 h after blocking the S-phase of the cell-cycle by treatment with a serum depleted-medium for 24 h. The cells were fixed in ice-cold 70% ethanol. Cells were incubated in PBS containing 40 μg/mL RNase A for 30 min at 37°C and resuspended in PBS containing 50 μg/mL propidium iodide. Analysis of the cell cycle was assessed by a BD FACScan Cytometer (Becton Dickinson, Franklin Lakes, NJ, USA).

### Cell proliferation assay, cell viability assay and cytotoxicity assay

The effects of NaB, TSA and VPA on cell proliferation were measured by quantifying BrdU (Bromodeoxyuridine) via a cell proliferation immunoassay from Roche Diagnostics (Mannheim, Germany). Twenty-four hours after seeding (1.5×10^4^/well, 96-well plates) cells were incubated with 10 μM BrdU and NaB, TSA and VPA at the indicated concentrations for 24 h. The incorporation of BrdU into proliferating cells was detected by using a peroxidase-conjugated antibody which reacts with the thymidine analogue BrdU and with BrdU incorporated into newly synthesized DNA. Bound anti-BrdU-peroxidase conjugated antibody was detected by a substrate reaction, and then quantified colorimetrically at 370 nm with a reference wavelength of 492 nm by an ELISA plate reader (ELISA-Reader ASYS Expert 96, Deelux Labortechnik, Gödenstorf, Germany). Cell viability was determined in LECs using alamar blue assay (Bio-Rad, Puchheim, Germany) which quantifies the metabolic conversion of resazurin to a highly fluorescent resorufin by viable cells. Twenty-four hours after seeding (1x10^5^/well, 96-well plates) cells were incubated with NaB, TSA and VPA at the indicated concentrations for 24 h. Alamar blue dye was added and the plates were incubated at 37°C for 6 h. The color change was measured fluorometrically at 530 nm excitation and 620 nm emission by using CytoFluor 4000 fluorometer (Perspective Biosystems, Framingham, MA, USA). The cytotoxic potential of TSA was determined using a lactate dehydrogenase-based cytotoxicity detection kit from Roche (Mannheim, Germany). Twenty-four hours after seeding, the cells were incubated with TSA for 24 h at the indicated concentrations.

### Short term lymphangiogenesis assay

Growth factor reduced Matrigel™ (BD Discovery Labware, Bedford, MA, USA) was placed into the lower chambers of μ-slide angiogenesis wells (IBIDI, Planegg/Martinsried, Germany) and hardened for 30 min at 37°C. Then, 1.5×10^4^ LECs/wells were seeded on the Matrigel and were either left untreated (solvent only, Ethanol) or incubated with TSA (at 400 nM for 6 h). Images were taken on the Biozero BZ-8000K microscope (Keyence Deutschland GmbH, Frankfurt am Main, Germany). Image acquisition was performed using BZ analyser software (Keyence Deutschland GmbH, Frankfurt am Main, Germany).

### Apoptosis assay

The effect of TSA on apoptosis was analysed using a Cell Death Detection ELISA PLUS-Kit from Roche Diagnostics. This assay detects DNA fragmentation that is characteristic of apoptotic cell death by quantifying mono-and oligonucleosomes in the cytoplasmatic fraction of cell lysates. The assay was carried out according to the manufacturers’ instructions. Briefly, synchronized cells were seeded in 96-well plates (1.5×10^4^ per well) and were exposed to 400 nM TSA for 24 h as indicated. Cell lysates were placed into a streptavidin-coated microtiter plate followed by the addition of anti-histone-biotin and anti-DNAperoxidase. The quantification of the amount of nucleosomes retained in the immunocomplexes was determined photometrically using 2.29-azino-bis-3-ethylbenzthiazoline-6-sulfuric acid as the substrate.

### Cytochrome c release

LECs were plated in cell culture dishes (5×10^6^ cells/60 cm^2^) and treated the next day with TSA as aforementioned. After 24 h, cytoplasmic extracts were obtained by digitonin permeabilization. Briefly, cells were trypsinized (0.125% trypsin/0.1% EDTA) for 5 min and centrifuged at 259 g for 10 min, and the pellet was resuspended in 250 μl PBS. Permeabilization of the membranes was obtained by adding 250 μl digitonin/sucrose (80 mg/ml, Fluka, Buchs, Switzerland) for 30 s. Then, samples were centrifuged for 1 min at 14000 g [12], and supernatants with equal amounts of protein (protein determination by DC protein assay; BioRad, Munich, Germany) were used in a commercial cytochrome c immunoassay kit (R&D Systems, Wiesbaden, Germany). The assay was performed according to the manufacturer’s manual. Optical density (450 nm) was measured using an ELISA reader (ELISA-Reader ASYS Expert 96, Deelux Labortechnik, Gödenstorf, Germany).

### Western blot analysis

Protein extracts were prepared as described previously [13]. Following SDS-PAGE and electroblotting, membranes were incubated with the following primary antibodies: anti-p21 (#2947, Cell Signaling, Danvers, MA, USA), anti-p27 (#3686, Cell Signaling, Danvers, MA, USA), anti-p53 (#9282, Cell Signaling, Danvers, MA, USA), anti-cleaved caspase 3 (#9661, Cell Signaling, Danvers, MA, USA), anti-cleaved caspase 7 (#9491, Cell Signaling, Danvers, MA, USA), anti-cleaved caspase 9 (#9501, Cell Signaling, Danvers, MA, USA), anti-cIAP-1 (#4952, Cell Signaling, Danvers, MA, USA), anti-cIAP-2 (#3130, Cell Signaling, Danvers, MA, USA), anti-tubulin (#3873, Cell Signaling, Danvers, MA, USA), anti-cyclin A (sc-271645, Santa Cruz, CA, USA), anti-cyclin D1 (sc-718, Santa Cruz, CA, USA), anti-CDK4 (sc-70831, Santa Cruz, CA, USA) and anti-CDK6 (sc-177, Santa Cruz, CA, USA). Primary antibody application was followed by incubation with horseradish peroxidase-conjugated secondary antibodies (anti-mouse and anti-rabbit IgG, Amersham, Uppsala, Sweden; anti-goat, Dako, Glostrup, Denmark). Blots were developed using an enhanced chemiluminescence detection system (Amersham) according to the manufacturer’s instructions.

### Immunolocalization of p21

To analyse the intracellular distribution of p21 in LECs, cells were seeded on glass coverslips in 24-well plates. The cells were grown in endothelial cell growth medium supplemented with 10% human serum for 24 h, fixed with methanol at −20°C and permeabilized with 0,1% TritonX-100 for 5 min at room temperature. Permeabilized cells were rinsed three times with PBS and incubated in blocking solution (1% bovine serum albumin/PBS) for 30 min at room temperature to remove nonspecific binding of the antibody. p21 was detected using a rabbit monoclonal anti-p21 antibody (#2947, Cell Signaling, Danvers, MA, USA) and an Alexa Fluor 546 goat anti-rabbit IgG secondary antibody. The slides were mounted in Aqua-Poly/Mount (Polysciences, Inc., Warrington, PA, USA) and viewed with fluorescence microscopy. Cells were counterstained for nuclei with Hoechst staining.

### Small interfering RNA

All small interfering RNA (siRNA) reagents including transfection reagents were obtained from Santa Cruz biotechnology (Santa Cruz, CA, USA). Cells were transfected with non-target siRNA (siRNA-A), used as negative control, p21 siRNA and p53 siRNA. The experiments were carried out following the manufacturer’s standard procedures. Total protein was extracted, and Western blot analysis was performed.

### RNA extraction and reverse transcription–PCR

Reverse transcription–PCR (RT-PCR) analyses were performed on total RNA (150 ng) extracted from subconfluent cell cultures. Total cellular mRNA was isolated by the RNeasy Mini Procedure (Qiagen, Hilden, Germany) after DNase digestion. RT-PCR analyses for p21, p27, p53 and β-Actin were performed with the One StepRT-PCR Kit (Qiagen, Hilden, Germany). PCR products were resolved by 1–2% agarosegel electrophoresis, and ethidium bromide-stained bands were visualized using an ultraviolet transilluminator.

### Real time PCR analysis

Total cellular RNA was isolated by the RNeasy Mini Procedure (Qiagen, Hilden, Germany) after DNase digestion. 750 ng of RNA was used for first strand cDNA synthesis using QuantiTect RT-Kit (Qiagen, Hilden, Germany). Real time PCR reactions were performed with the SYBRgreen dye technique on a Light Cycler system (Roche Diagnostics, Mannheim, Germany).

### Transient transfection and analysis of reporter gene expression

Human lymphatic endothelial cells (0.8×10^5^/well, 12-well plates) were transfected with 1 μg of an appropriate firefly luciferase construct and 0.5 μg of phRG-TK vector (Promega, Mannheim, Germany) using the SuperFect transfection reagent (Qiagen, Hilden, Germany). Human p21 reporter gene constructs were described elsewhere [14]. Twenty-four hours after transfection, control transfectants were left untreated and test transfectants were exposed to 400 nM TSA for 24 h. The activities of luciferases were measured utilizing the Dual-Luciferase Reporter Assay System from Promega (Mannheim, Germany).

### Preparation of nuclear extracts and gel mobility-shift analysis

Human lymphatic endothelial cells were untreated (solvent only, Ethanol) or were incubated with 400 nM TSA for 60 min. Nuclear proteins were extracted as described previously [15]. The DNA-binding reactions were performed by using nuclear extracts (3.5 μg) and biotin-labeled DNA probes with or without a competitive cold DNA probe, a Sp1 and Sp3 antibody (Santa Cruz, CA, USA). Protein-DNA complexes were detected by streptavidin-HPR and ECL (Signosis, Santa Clara, CA, USA).

### Statistical analysis

Data are expressed as mean ± SE from ≥3 independent experiments. Statistical analysis was performed by a Student’s t-test.

## Results

### Effects of HDACi on cell proliferation, cell viability and in vitro lymphangiogenesis in human lymphatic endothelial cells (LEC)

**Fig. 1:**
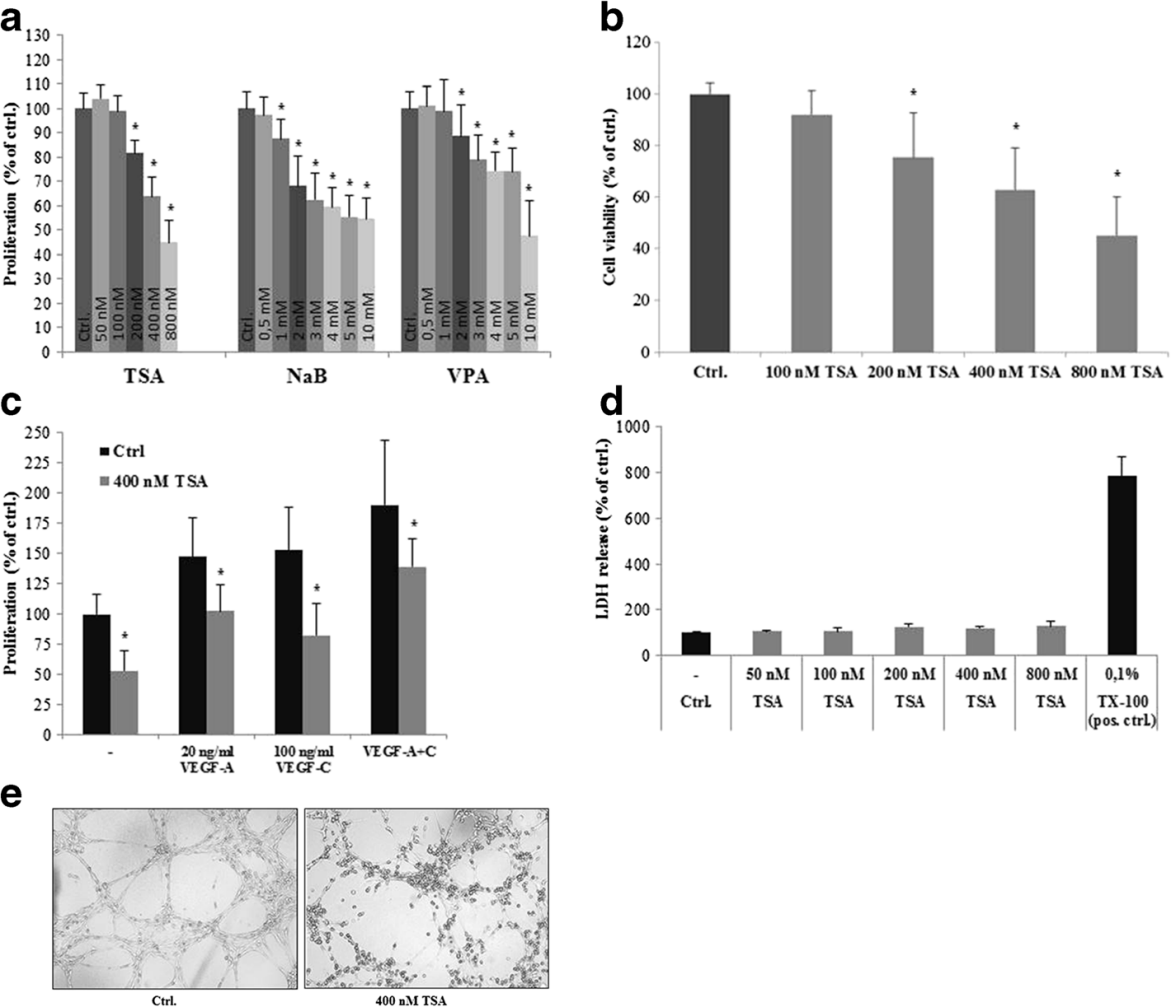
Effects of HDACi on primary human lymphatic endothelial cells. **a**, **b** Cells were exposed to increasing concentrations of trichostatin A (TSA), sodium butyrate (NaB) and valproic acid (VPA) alone and (**c**) in the presence or absence of 20 ng/ml VEGF-A and 100 ng/ml VEGF-C for 24 h as indicated. Cell proliferation and viability was measured using the BrdU (**a**, **c**) and Alamar blue assay (**b**). Average absorbance values (mean ± SE) from 4 wells per experimental condition are displayed; data are expressed as cell proliferation and vialibity in percentage (%) with regard to solvent controls (=100%; ethanol and H_2_O). Results were confirmed in four independent sets of experiments. *p < 0.05 vs Ctrl. **d** Quantification of cytotoxicity. Cells were incubated with increasing concentrations of TSA for 24 h as indicated. Cytotoxicity was quantified by using the LDH assay. Average absorbance values (mean ± SE) from quadruplicate determinations per experimental condition were calculated; data are expressed as cytotoxicity in percentage (%). **e** A two-dimensional, short term in vitro Matrigel assay of LECs that were left untreated or were incubated with 400 nM TSA for 6 h on Matrigel. Results were confirmed in three independent sets of experiments

We investigated the impact of HDACi on lymphangiogenesis in primary human lymphatic endothelial cells in vitro. First, we tested the effects of different HDACi (NaB, TSA and VPA) on cell proliferation and cytotoxicity using human primary lymphatic endothelial cells (LECs). In our analyses we found that HDACi inhibited cell proliferation and viability in a concentration-dependent manner, as determined by the BrdU and alamar blue assay. Interestingly, these effects could not be abrogated by the addition of the pro-lymphangiogenic growth factors VEGF-C and -A, indicating that HDACi could influence angiogenic signaling pathways (Fig. 1a, b). To determine the cytotoxic effects of TSA, a potent pan-HDACi, we performed lactate dehydrogenase assays and found no cytotoxic effects (Fig. 1c). Furthermore, we could demonstrate, by using a matrigel assay, that TSA inhibited capillary-like structure formation (Fig. 1d). These data suggest that HDACi have distinct anti-lymphangiogenic effects by affecting lymphatic endothelial cell functions.

### TSA induces G0/G1 cell cycle arrest in LECs

**Fig. 2:**
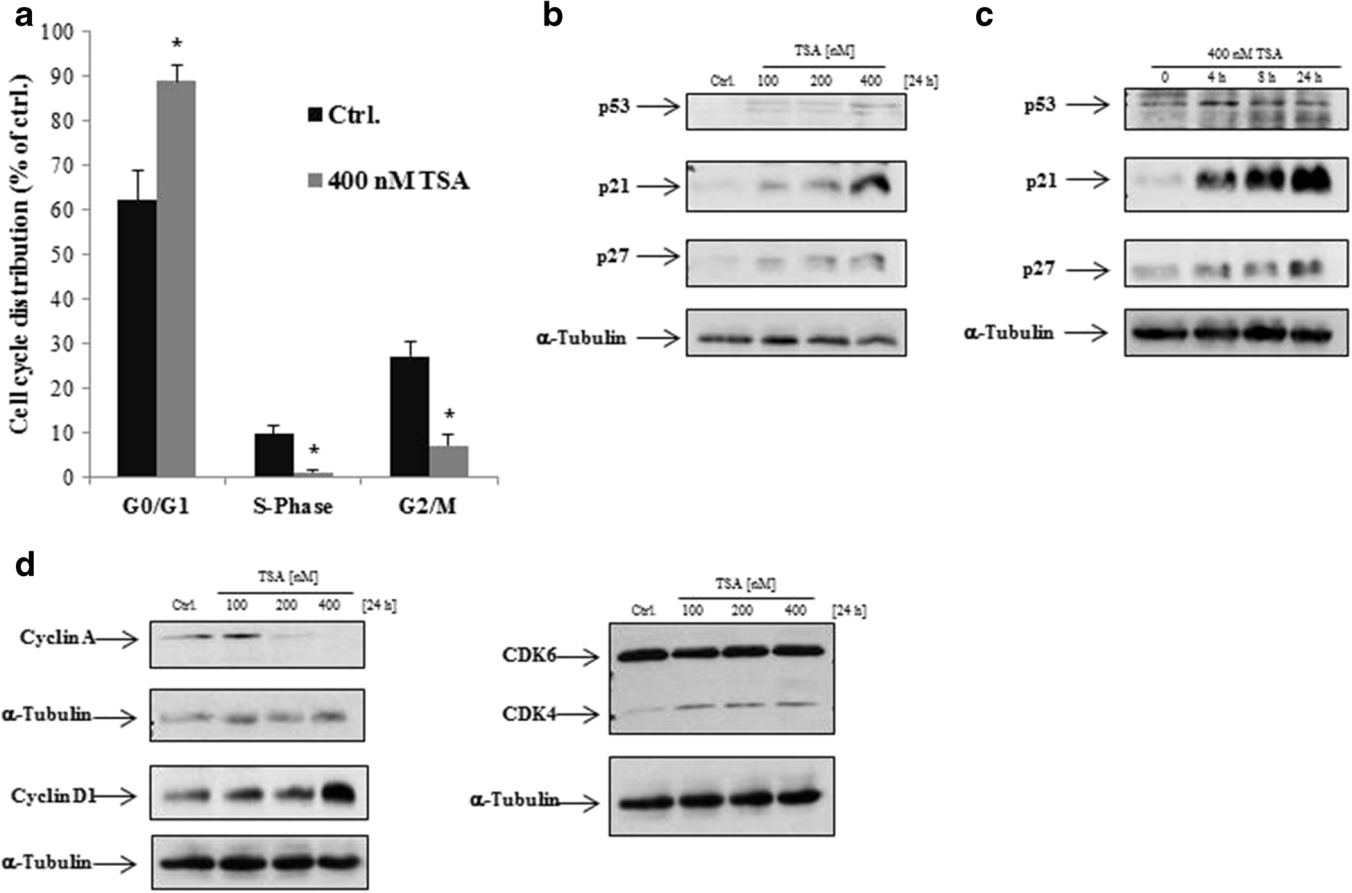
TSA induces G0/G1 cell cycle arrest in LECs. **a** Cells were incubated with 400 nM TSA and ethanol as a control for 24 h after blocking the S-phase of the cell-cycle by treatment with a serum depleted-medium for 24 h. Analysis of cell cycle as assessed by FACS using propidium iodide-stained LECs. The figure shows the percentage of cells in G0/G1, S and G2/M with regard to solvent controls (Ethanol). Data displayed are representative of eight experiments that were performed revealing comparable results. *p < 0.05 vs Ctrl. **b**, **c** and **d** Representative western blot analyses of LECs that were left untreated (solvent only) or were treated with TSA in a concentration- and time-dependent manner. Cell cycle control related proteins, like p21, p27, p53, cyclin A/D1, CDK4, CDK6 and α-Tubulin as loading control were detected by enhanced chemiluminescence. Comparable results were obtained from three independent experiments

To investigate the major underlying mechanisms of HDACi-induced growth inhibition of LECs, we next analysed the cell cycle distribution using flow cytometry. We found that the percentage of cells in the G0/G1 phase was significantly increased upon TSA treatment for 24 h (Ctrl.: 62.22 ± 6.83%; 400 nM TSA: 89.03 ± 3.51%). In addition, the percentage of cells in G2/M- (Ctrl.: 27.19 ± 3.18%; 400 nM TSA: 7.29 ± 2,28%) and S-Phase (Ctrl.: 9.72 ± 2.03%; 400 nM TSA: 1.14 ± 0.43%) was significantly reduced after treatment with TSA (Fig. 2a). Next, we analysed the effects of TSA on important G0/G1-cell cycle regulators. Interestingly, a report showed that HDACi does not induce expression of the important cell cycle regulators p21 and p27 in an artificial lymphatic-like endothelial cell line (named FP01) [11]. As shown in Fig. 2b and Additional file 1, TSA induced the protein expression of p21, p27 and p53 in a concentration-dependent and time-dependent manner (Fig. 2c). Surprisingly, we observed an upregulation of cyclin D1 and Cdk4 after treatment with TSA. On the other hand, since the cells were blocked in the G0/G1-phase, expression of cyclin A was decreased after treatment with TSA while the expression of Cdk6 was unaffected (Fig. 2d). In summary, these results demonstrated that HDACi-induced G0/G1-cell cycle arrest in LECs is due to modulating of the expression of cell cycle regulating proteins.

### TSA induces apoptosis through activation of the intrinsic pathway

**Fig. 3:**
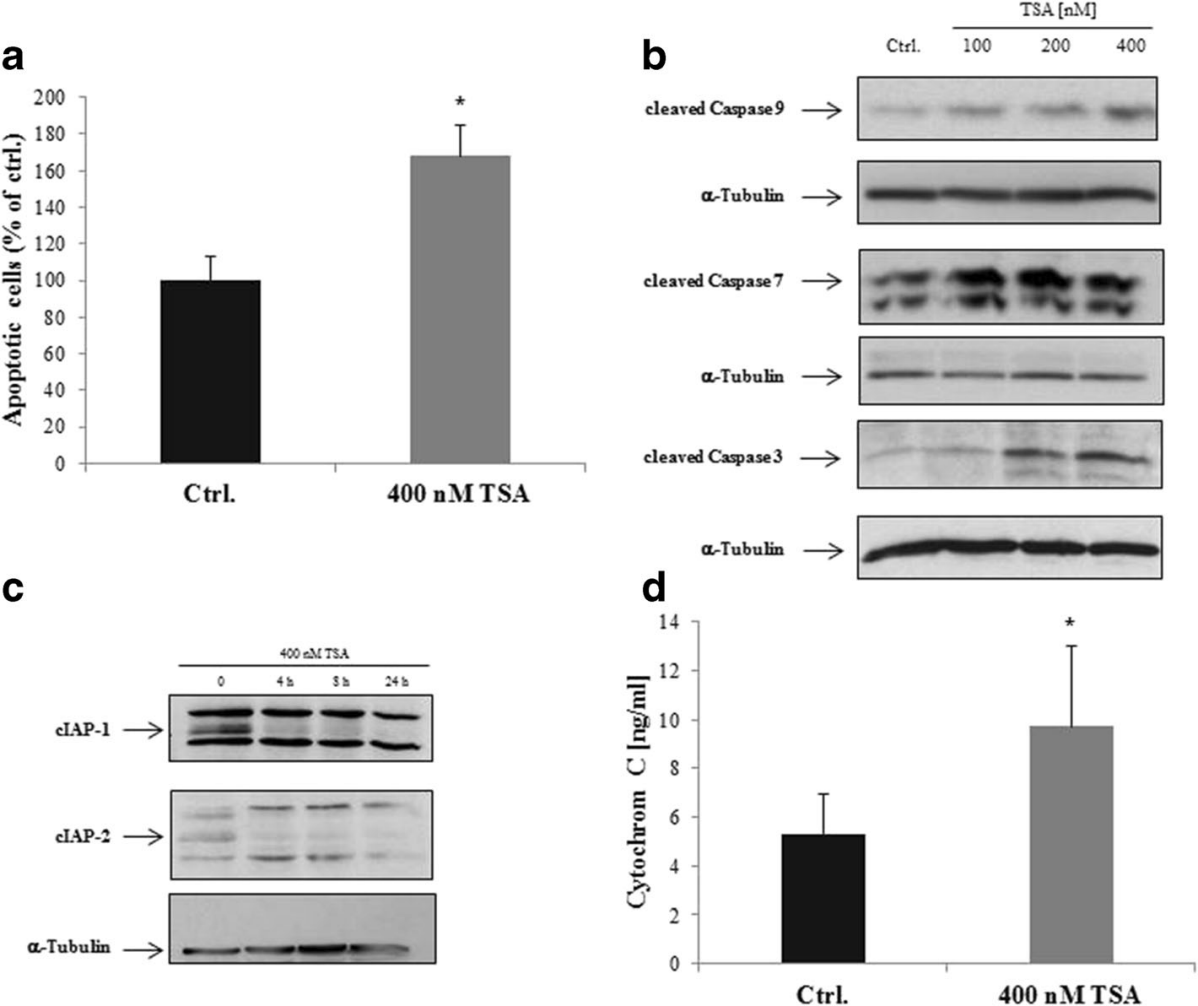
TSA treatment induces the intrinsic apoptotic pathway in LECs. **a** Cells were exposed to 400 nM TSA for 24 h as indicated. For apoptosis determination, we used a colorimetric assay that quantified histone complexed DNA fragments. Average absorbance values (mean ± SE) from 3 wells per experimental condition are displayed; data are expressed as apoptotic cells in percentage (%) with regard to solvent controls (=100%; ethanol). *p < 0.05 vs Ctrl. **b** and (**c**) Representative western blot analyses of LECs that were left untreated (solvent only) or were treated with TSA in a concentration- and time-dependent manner. Apoptosis related proteins, like cleaved caspases 9, 3 and 7, cIAP-1/2 and α-Tubulin as loading control were detected by enhanced chemiluminescence. Comparable results were obtained from three independent experiments. **d** LECs were left untreated (solvent only, Ethanol) or were treated with 400 nM TSA for 24 h. The release of cyctochrome c to cytosol was measured using the cytochrome c ELISA. Mean values from four independent experiments are depicted ± SE (error bars). *p < 0.05 vs Ctrl

We next analysed the impact of TSA on apoptosis in LECs. Figure 3a shows that TSA induced potent apoptosis in LECs up to 168% compared with untreated controls. It has been found that HDACi induce apoptosis in various cell types (e.g. breast cancer cells, melanoma cells and cervix carcinoma cells) by activating caspase-dependent pathways and regulating different pro- and anti-apoptotic genes [16]. Therefore, we analysed the effects of TSA on apoptosis-related proteins in LECs. As shown in Fig. 3b and Additional file 2, increased cleavage of caspases 3, 7 and 9 was observed after a 24 h exposure to TSA. Furthermore, we could demonstrate that the expression of anti-apoptotic proteins cIAP-1 and −2 was decreased already after 4 h treatment with TSA (Fig. 3c). To determine whether HDACi induced the intrinsic pathway in LECs, we analysed the release of cytochrome c from mitochondria after treatment with TSA (Fig. 3d). We found that TSA induced a release of cytochrome c after a 24 h treatment, indicating that the HDACi TSA might activate the intrinsic apoptotic pathway in LECs. These results demonstrated that TSA induces the intrinsic apoptotic pathway by cytochrome c release, activating caspases 3, 7 and 9 and downregulating the anti-apoptotic proteins cIAP-1 and −2.

### TSA causes nuclear accumulation of p21

**Fig. 4:**
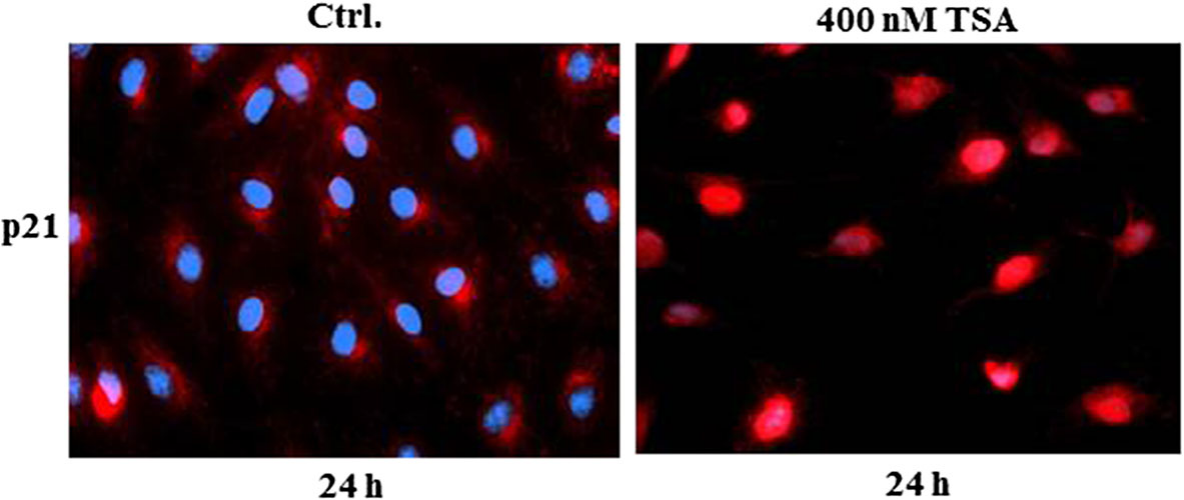
TSA mediates nuclear accumulation of p21 protein in LECs. Subconfluent LECs were either left untreated (solvent only) or treated with 400 nM TSA for 24 h. Cells were fixed and stained with anti-p21 antibody. *Blue*: Hoechst stained nuclei; *red*: p21 protein stained with specific antibody and secondary antibody conjugated with Alexa Fluor 546. Comparable results were obtained from three independent experiments

Several studies have indicated that p21 plays a critical role in the modulation of apoptosis. It is well known that p21 regulates cell cycle progression and apoptosis depending on its subcellular distribution [17, 18]. To investigate whether TSA induced nuclear accumulation of p21 in LECs, we analysed the subcellular distribution of p21 after treatment with TSA by immunofluorescence staining. In our experiments we observed that accumulated p21 protein was found to be located in nuclei of the TSA treated LECs (Fig. 4). These data suggest that TSA may induce pro-apoptotic and anti-proliferative effects in LECs by mediating nuclear accumulation of p21.

### TSA upregulate the steady state of p21 mRNA

**Fig. 5:**
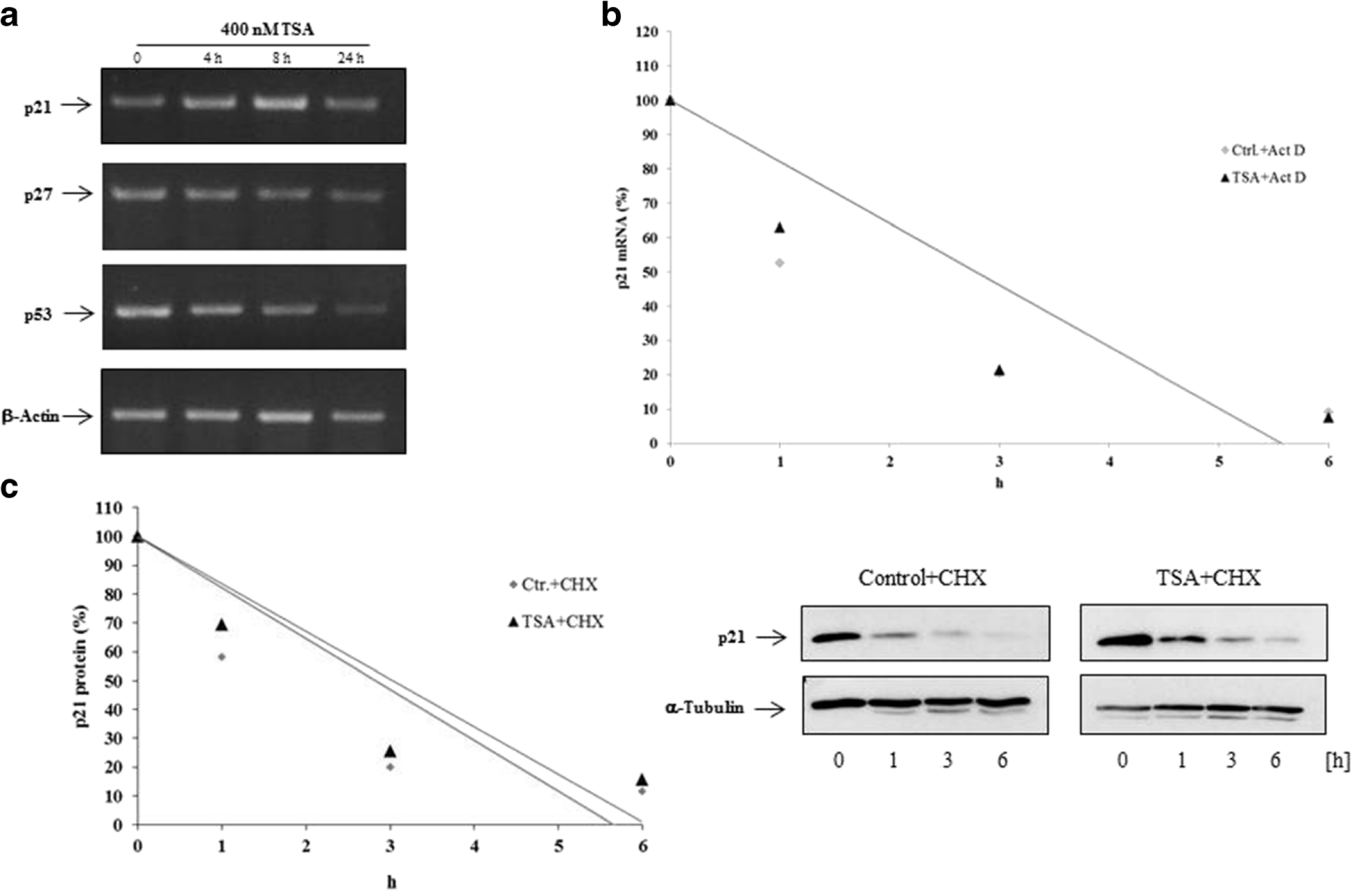
p21, but not p27 and p53, mRNA expression is upregulated by TSA. **a** Semiquantitative RT-PCR analyses for p21, p27, p53 and β-Actin as loading control were performed on total RNA extracted from subconfluent LECs. LECs were left untreated (solvent only, ethanol), or were treated with TSA (at 400 nM) for the indicated times. Results were confirmed in three independent sets of experiments. **b** LECs were incubated in the presence or absence of TSA (400 nM) for 3 h and incubated for 0, 1, 3 and 6 h with fresh media containing actinomycin D (10 μg/ml). Then, p21 mRNA levels were quantified using quantitative PCR. Comparable results were obtained from three independent experiments. **c** LECs were incubated in the presence or absence of TSA (400 nM) for 3 h and incubated for 0, 1, 3 and 6 h with fresh media containing cycloheximide (10 μg/ml). p21 and α-tubulin protein were detected by enhanced chemiluminescence. p21 protein remaining at these time points was determined by densitometric scanning, the results of which were normalized to amounts of α-tubulin. Comparable results were obtained from three independent experiments

Recent studies suggested that HDACi induced p21 expression in various cancer cell lines in a transcriptional manner [19]. To investigate further the mechanism of HDACi-induced p21, p27 and p53 expression in human primary lymphatic endothelial cells, we performed RT-PCR analysis after treatment with TSA. Our data demonstrate increased p21 mRNA expression in a time-dependent manner while the expression of p27 and p53 was downregulated (Fig. 5a). Several studies revealed that HDACi strongly induce the expression of p21 in many cancer cell lines in a transcriptional, posttranscriptional, and posttranslational manner [20, 21]. We therefore first analysed a possible posttranscriptional and posttranslational mechanism of control. To verify these assumptions, we first investigated p21 mRNA and protein stability in vehicle- and TSA-treated LECs. In the presence of actinomycin D or cycloheximide, p21 mRNA and protein stability remained unchanged in TSA-treated cells, which argues against any posttranscriptional or -translational mechanism of p21-induction after HDACi-treatment (Fig. 5b, c).

### TSA increases p21 gene transcription through Sp1/3 binding sites

**Fig. 6:**
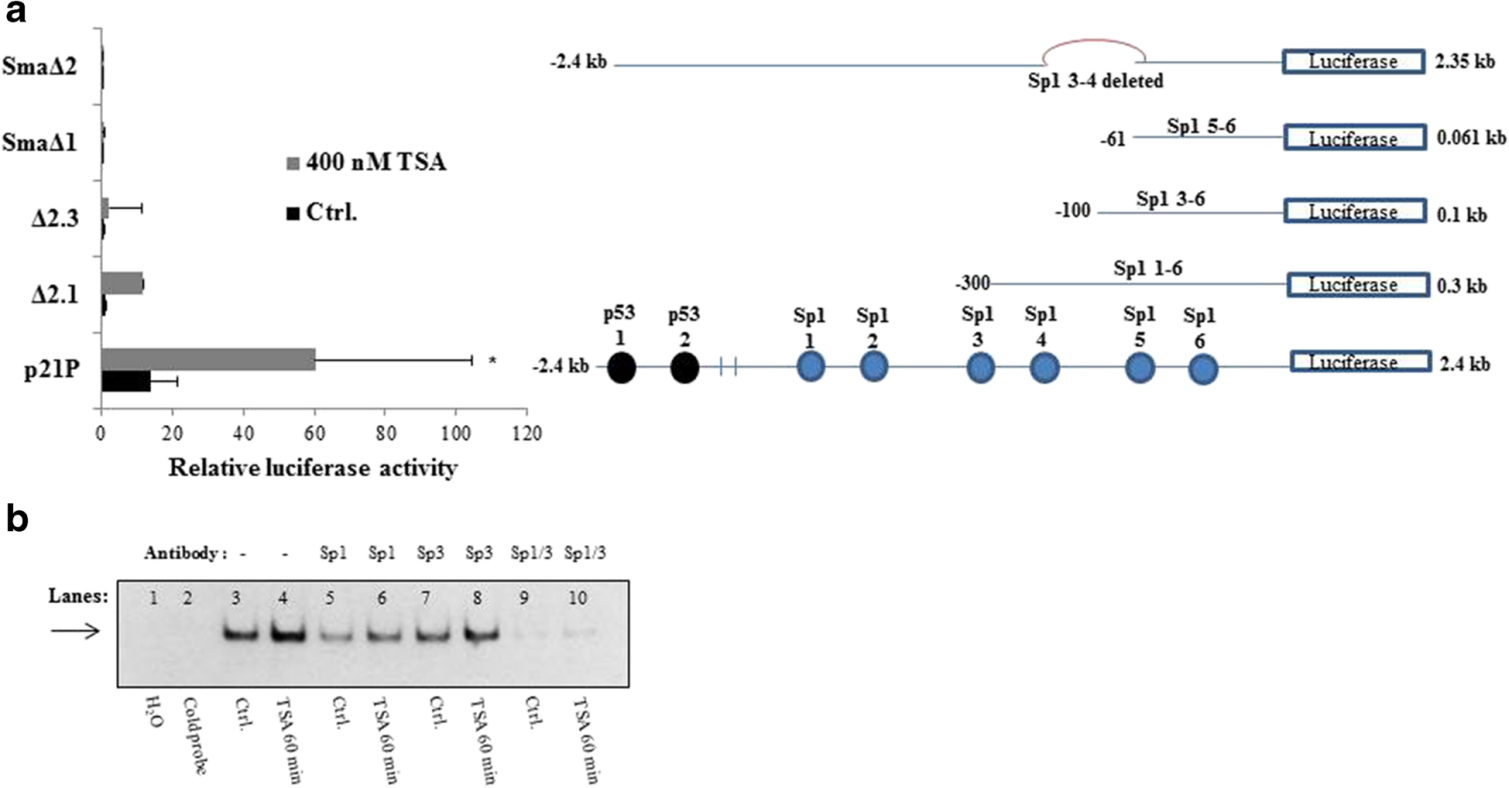
TSA induces p21 promoter activity via increased Sp1/3-dependent binding to the −100/–81 bp sequence. **a** Analyses of 5’-deletional p21 promoter-based luciferase (Luc) constructs in LECs. Schematic representation of the respective reporter gene constructs on the right and the relative Luc activities (expressed as % basal activity of the −2325/+8 construct) in graphic format on the left. *Black* bars, untreated controls (Ctrl.); grey bars, TSA-treated cells (mean ± SE of three independent duplicate assays). *p < 0.05 vs Ctrl. **b** Representative EMSA using nuclear extracts of untreated (Ethanol only) and TSA-treated (at 400 nM, 1 h) LECs. The DNA-binding activity of Sp1/3 was measured by using nuclear extracts and biotin-labeled DNA probes with or without a competitive cold DNA probe. Supershift experiments were carried out by incubating nuclear extracts with Sp1/3 antibodies. The formation of Sp-dependent binding complexes is indicated by arrows to the left

Several studies indicated that p53 and Sp1/3 binding sites of the p21 promoter are critical for p21 induction by HDACi treatment [22–24]. To determine the function of p53 and Sp1/3 transcription factors in the activation of p21 promoter activity after treatment with HDACi, we next performed luciferase promoter assays. Luciferase reporter constructs containing the 5`-region of the p21 promoter and a series of deletional constructs were transiently transfected into solvent- and TSA-treated LECs. We could demonstrate that TSA incubated cells have shown 4.5-folds of p21 promoter activity. Furthermore, we could show that the p21-Δ2.1 vector, which lacks the two p53-binding sites but contains six Sp1/3-binding sites, was still activated after TSA-treatment. To determine which Sp1/3-binding sites of the promoter region of the p21P (=human full length promoter) vector are responsive to HDACi, we tested a series of deletional constructs, lacking important Sp-binding sites. A deletion in the Sp1-2 binding site (Δ2.3) did not change the promoter activity after TSA-treatment. In addition, deletion of the Sp1-4 (SmaΔ1) and Sp3-4 (SmaΔ2) binding site abolished completely p21 promoter activity after exposure to HDACi (Fig. 6a). In summary, we could demonstrate that the Sp3-4 binding site in the region −100 to −81 bp contains the main responsive elements for transcriptional p21 activation by HDACi-treatment in primary human lymphatic endothelial cells. To determine which nuclear factors bind to the Sp1/3 binding site, we performed electromobility shift assays using nuclear extracts from LECs and biotin-labeled DNA probes. In untreated cells, a distinct complex was observed to bind (Fig. 5b, lane 3). We could find a significant increase in DNA binding activity in cells exposed to TSA (Fig. 6b, lane 4). A decreased binding activity was found upon addition of Sp1 antibody (Fig. 6b, lane 6) or an Sp3 antibody (Fig. 6b, lane 8). DNA binding activity was almost completely abolished in the presence of Sp1/3 antibodies (Fig. 6b, lane 10). Competition assays using an excess amount of unlabeled oligonucleotide (Fig. 6b, lane 1) revealed that nuclear proteins bind to the p21 promoter sequence in a Sp1/3-dependent manner (Fig. 6b, lanes 6 and 8).

### The pan-HDACi TSA mediates its anti-proliferative effects on LECs through p21 dependent pathways

**Fig. 7:**
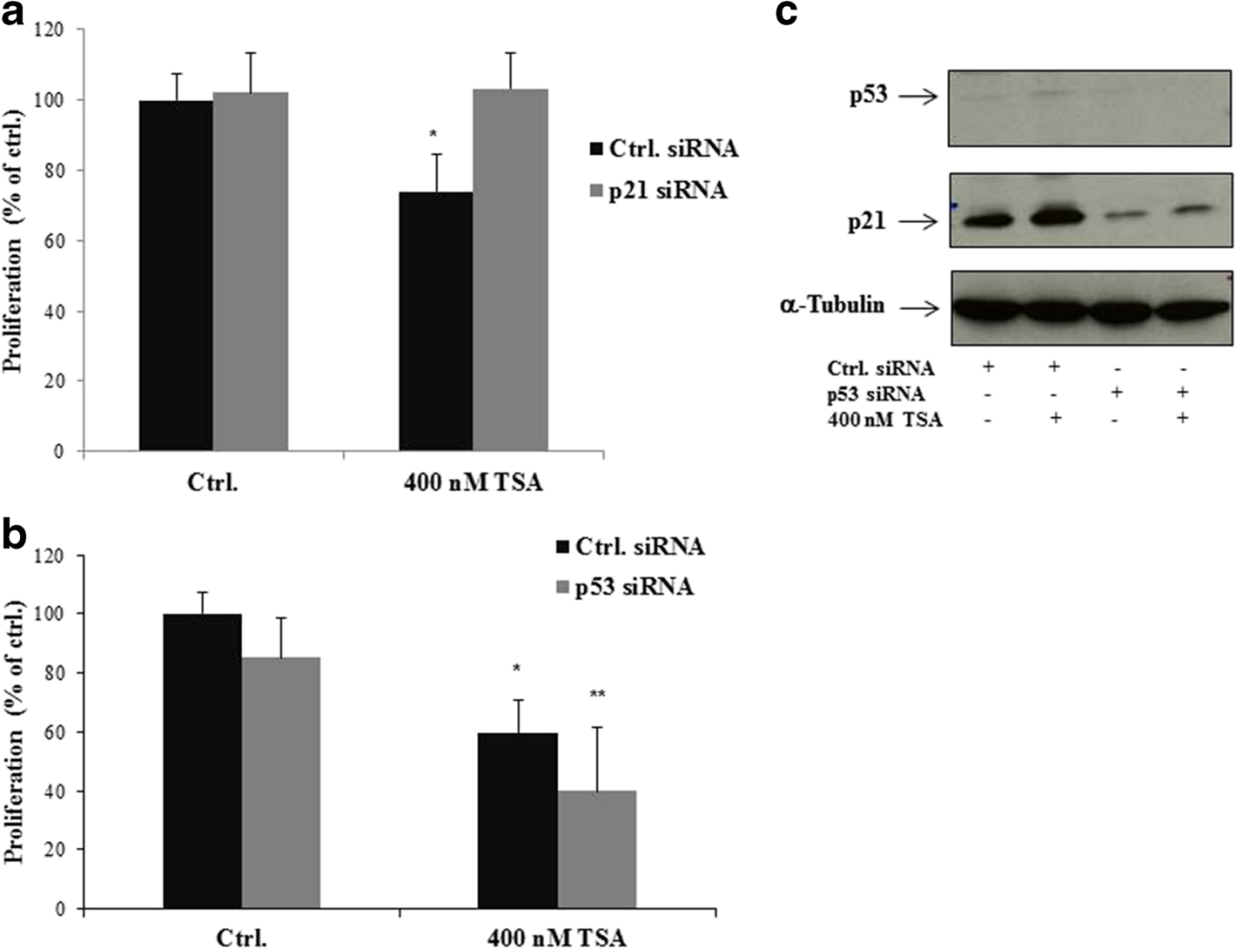
The pan-HDACi TSA mediates its anti-proliferative effects on LECs through p21 dependent pathways. **a** Cells were treated with siRNA against p21, p53 (**b**) and control siRNA and were exposed to 400 nM TSA or solvent only (=Ethanol; 100 %) for 24 h as indicated. Cell proliferation was measured using the BrdU assay. Average absorbance values (mean ± SE) from 3 wells per experimental condition are displayed; data are expressed as cell proliferation in percentage (%) with regard to solvent controls (=100%; ethanol). *^,^**p < 0.05 vs Ctrl. **c** Representative Western blot analyses of LECs that were incubated with siRNA against p53 or control siRNA and treated with TSA (at 400 nM) or solvent only (Ethanol) as indicated for 24 h. p53, p21 and α-Tubulin protein as loading control were detected by enhanced chemiluminescence. Comparable results were obtained from three independent experiments

To examine the role of p21 and p53 on TSA-mediated anti-proliferative effects in LECs, we performed siRNA knockdown assays. Our data demonstrate that knockdown of p21 effectively reversed the TSA-induced growth inhibition of LECs (Fig. 7a, Additional file 3) whereas silencing of p53 showed no effects on cell proliferation (Fig. 7b, Additional file 3). We also analysed the effect of p53 silencing on TSA-induced p21 expression in LECs. The knockdown of p53 by siRNA in LECs did not influence the upregulation of p21 induced by TSA (Fig. 7c, Additional file 4). In summary, we could demonstrate that p21 is essential for TSA-mediated growth inhibition in LECs. Furthermore, we found that p53 is dispensable for TSA-induced p21 protein expression in LECs.

## Discussion

Lymphangiogenesis is an essential step in the initiation and progression of cancer. The presence of metastasizing tumour cells in regional lymph nodes is one of the key predictors of poor outcome in various tumour entities. It has been found that intratumoural lymphatics in melanoma are associated with distant metastasis and poor disease free survival [10]. Further studies have shown that enhanced lymphatic invasion in melanoma is associated with sentinel lymph node metastasis and is a prognostic indicator of distant metastasis [25, 26]. Since HDACs regulate different cell functions involving cell survival, cell cycle progression and differentiation, they are recognized as promising targets for cancer therapy [3]. HDACi have recently been shown to mediate anti-angiogenic activities in endothelial cells by various mechanisms, including downregulation of pro-angiogenic factors such as VEGFR1, VEGFR2, Neuropilin-1, Tie-2 and HIF-1α as well as upregulation of factors which inhibit angiogenesis, such as p53 and von Hippel-Lindau. In addition, it has been reported that various HDACi impaired angiogenesis in vitro and in vivo [27–30]*.* These results suggest the growing importance and the possible applicability of HDACi for anti-angiogenic therapy. Our data provide evidence that HDACi have distinct anti-lymphangiogenic effects in part due to the suppression of proliferation and tube formation of primary human lymphatic endothelial cells. Several studies recognized HDACi as promising agents for anti-lymphangiogenic therapy. Yamamura et al*.*, demonstrated that NaB inhibited pro-lymphangiogenic factors (e.g. VEGF-C and angiopoietin-2) in the oral cancer cell line HSC-3 [31]. Furthermore, Cheng and Hung found that SAHA, a potent HDACi, inhibits VEGF-C expression in breast cancer cells via Sp1-dependent transcriptional repression [32]. To date, however, little is known about the effects of HDACi on primary human lymphatic endothelial cells. There is only one study by Cheng et al. investigating the role of HDACi on lymphangiogenesis in an artificial immortalized lymphatic cell line, generated by transfection of the endothelial cell line EA.hy926 with PROX-1 [11]. Immortalized endothelial cells have been shown to exhibit differences relative to their primary cell counterparts. Recently, Boerma M et al. demonstrated that statins differentially regulated genes involved in cell proliferation, cell cycle regulation and DNA replication in immortalized endothelial cells (EA.hy926) compared to in primary human endothelial cells, making them less suited for studies focused on genes, which are involved in the regulation of cell proliferation and apoptosis [33]. The authors of the mentioned manuscript demonstrated that SAHA - a potent, reversible pan-HDACi - repressed lymphangiogenesis in the artificial FP01 cell line mimicking lymphendothelial cells. They found that SAHA inhibited proliferation, impaired sprouting, tube formation and cell cycle progression at the G0/G1 and G2/M phases. In a comparable manner, we found that HDACi inhibited proliferation of primary lymphatic endothelial cells, impaired tube formation and induced G0/G1-arrest. In the study of Cheng and colleagues, the expression of the important cell cycle inhibtors p21^WAF1/KIP1^, p27 ^KIP1^ and cyclin A was not changed by SAHA treatment, whereas cyclin D1 and B1 were increased and cyclin E was reduced. In contrast to the study of Cheng et al., we showed an effective up-regulation of the cell cycle inhibitors p21^WAF1/KIP1^, p27 ^KIP1^ and p53 by HDACi. In addition, we provide solid evidence that cell cycle arrest at the G0/G1-phase was accompanied by an inhibition of cyclin A and up-regulation of cyclin D1. Furthermore, our data indicate that HDACi induce p21^WAF1/KIP1^ expression by transcriptional activation of the promoter via Sp1/3 binding sites. In contrast to our study, Cheng and colleagues focused their study on Tie-2 signaling and observed that SAHA impaired sprouting and tube formation of the FP01 cell line by downregulating Tie-2 via transcriptional and post-transcriptional mechanisms [11]. In addition, we showed in our primary lymphendothelial cells that HDACi induced apoptosis by activating the intrinsic apoptotic pathway. Taken together, the in part contrary results of Cheng and colleagues may be explained by another experimental setting using an immortalized lymphatic endothelial cell line in contrast to primary lymphendothelial cells which are the standard in lymphangiogenesis research. Several studies demonstrated that HDACi can augment the extrinsic apoptotic pathway by upregulating of cell surface death receptors and/or ligand expression (e.g. Fas and TRAIL) causing activation of caspase-8 [34–36]. In addition, studies have demonstrated that HDACi induce the intrinsic apoptotic pathway in various cancer cell lines by disruption of the mitochondrial membrane, causing cytochrome c release and subsequent activation of the caspases-3, −7 and −9 [37–39]. Increasing evidence has revealed that HDACi induce the mitochondrial pathway by downregulating certain anti-apoptotic proteins, such as Bcl-2, Survivin and Bcl-xL, and upregulating pro-apoptotic proteins, such as Bad, Bid, Bim and Bmf [40–42]. In the present study, we demonstrate that TSA induced the intrinsic apoptotic pathway in primary human lymphatic endothelial cells by cytochrome c release, activating caspases-3, -7 and -9 and causing downregulation of the anti-apoptotic proteins cIAP-1 and −2. These results suggest that the induction of the intrinsic apoptotic pathway by TSA may be at least one mechanism responsible for the growth inhibition of primary human lymphatic endothelial cells and the subsequent anti-lymphangiogenic properties of HDACi. We provide solid evidence that TSA induces apoptosis in primary human lymphatic endothelial cells through activating the intrinsic apoptotic pathway. Further studies have demonstrated that HDACi suppress cancer cell growth by inducing cell cycle arrest at the G0/G1 and/or G2/M phases [43–47]. Almost all HDACi have been reported to activate the important cyclin-dependent kinase (CDK) inhibitor p21^WAF1/KIP1^. Numerous studies have provided evidence indicating that activation of p21^WAF1/KIP1^ is required for the induction of G0/G1 arrest and/or apoptosis in several cancer models by HDACi [48, 49]. Activation of p21^WAF1/KIP1^ after HDACi treatment is mainly accompanied by enhanced histone acetylation within the p21^WAF1/KIP1^ promoter region and increased binding of Sp1/3 transcription factors and/or p53 on the promoter [19–22, 50, 51]. Furthermore, several studies have shown that increased p21^WAF1/KIP1^ expression leads to repression of cyclin D and cyclin A, which contributes to the loss of CDK 4 and 6 kinase activity and the presence of hypophosphorylated retinoblastoma protein, subsequently inducing G0/G1 cell cycle arrest [52–54]. On the other hand, there is growing evidence that nuclear localized p21^WAF1/KIP1^ contributes to cell cycle arrest and control of DNA replication, whereas cytoplasmic distribution of p21^WAF1/KIP1^ has been suggested to inhibit apoptosis [17]. It has been found that cytosolic p21^WAF1/KIP1^ effectively blocks procaspase-3 mediated cell death in a human liver cancer cell line and that this interaction occurs on the mitochondria, suggesting the pivotal role of cytoplasmic p21^WAF1/KIP1^ in inhibition of apoptosis in cancer cell lines [55, 56]. In the study of Asada and colleagues, it has been demonstrated that cytosolic p21^WAF1/KIP1^ interacts as negative regulator of apoptosis by binding and inhibiting the pro-apoptotic kinases ASK1 and JNK in monocytes [57]. Several studies revealed that cytosolic p21^WAF1/KIP1^ has been related to drug resistance in various cancer cell types. Vincent et al. found that expression of Nuclear Protein 1 (NUPR1), a transcriptional regulator of p21^WAF1/KIP1^ expression, mediates growth benefit and chemoresistance by causing Akt-mediated phosphorylation, subsequent cytoplasmic relocalization of p21^WAF1/KIP1^ and activation of the anti-apoptotic Bcl-xL protein in breast cancer cells [58]. Furthermore, Xia et al. found that p21^WAF1/KIP1^ translocation into the cytoplasm via constitutively active Akt2 transfection in ovarian cancer cells enhanced the resistance to paclitaxel, while inhibition of p21^WAF1/KIP1^ translocation into the cytoplasm via Akt2 shRNA transfection in ovarian cancer cells significantly increased paclitaxel treatment sensitivity. Additionally, they demonstrated that knockdown of cytoplasmic p21^WAF1/KIP1^ by siRNA in Akt2 overexpressed cancer cells notably increased paclitaxel-induced apoptosis [59]. A further study showed that the HDACi NaB induced G1-cell cycle arrest and apoptosis in breast cancer cells. It has been shown that cell cycle arrest and apoptosis were accompanied with increased expression of p21^WAF1/KIP1^ protein and its accumulation in cellular nuclei. Interestingly, depletion of p21^WAF1/KIP1^ by antisense did not modified NaB-induced cell cycle arrest, whereas NaB-induced apoptosis was abolished in a PCNA-dependent manner. These results demonstrated that p21^WAF1/KIP1^ also possesses pro-apoptotic functions independent of cell cycle regulation [60]. Several other studies observed that nuclear accumulation of p21^WAF1/KIP1^ has been associated with pro-apoptotic effects [61, 62]. Taken together the role of p21^WAF1/KIP1^ on apoptosis may rely on different intracellular distribution. In this study, we demonstrate that TSA leads to cell cycle inhibition at the G0/G1 phase with a marked rise in p21^WAF1/KIP1^, p27^KIP1^ and p53 expression in human primary lymphatic endothelial cells. Furthermore, we found that p21^WAF1/KIP1^ accumulated in cellular nuclei due to TSA treatment, which may explain in part the growth inhibiting effects of p21^WAF1/KIP1^ in LECs. In addition, we observed that cyclin A was downregulated by TSA treatment, whereas CDK6 was unaffected after HDACi treatment. Interestingly, cyclin D1 and CDK4 were upregulated after TSA treatment, suggesting that the block in the G0/G1-phase due to HDAC inhibition was mediated by the cyclin-dependent kinase inhibitors p21^WAF1/KIP1^, p27^KIP1^, p53 and by downregulation of cyclin A. Other studies have reported a cyclin D-independent G1 cell cycle arrest. It has been reported that the HDACi NaB induces G1-arrest in colon adenocarcinoma cells by stimulating cyclin D and p21^WAF1/CIP1^ and inhibiting CDK2 expression [63]. In addition, studies have demonstrated transient overexpression of cyclin D1 arrested fibroblasts and HC11 mouse mammary epithelial cells in the G1-phase [64, 65]. At the present, it is not fully clear whether cyclin D mediates any CDK-independent mechanisms in cell cycle arrest. It is generally accepted that p21^WAF1/KIP1^ gene expression is regulated by p53 binding sites localized upstream of the coding sequence (>1.9 kb) and a proximal promoter sequence consisting of six Sp1 binding sites [66]. Since TSA is known to induce p21^WAF1/KIP1^ through increased binding of Sp1/3 transcription factors and/or p53 on the promoter [19], we hypothesized that the p21^WAF1/KIP1^ promoter should be activated by the increased binding of transcription factors. It has been observed that TSA induces p21^WAF1/KIP1^ expression in a human T cell leukaemia cell line via transcriptional mechanisms through the Sp1 site in its promoter [50]. A study showed that Sp3 mediates transcriptional activation of the p21^WAF1/KIP1^ gene promoter in osteosarcoma cells by HDAC inhibition [67]. Consistent with these data, we observed that TSA activates p21^WAF1/KIP1^ transcription in primary human lymphatic endothelial cells via Sp1/3 binding sites in the proximal promoter (between −100 and −80 bp). Recent studies revealed that HDACi induce growth arrest in several cancer cell lines in a p53-dependent and independent way [68–70]. In our study, we observed that depletion of p53 does not influence the expression status of p21 induced by HDACi, indicating that TSA mediated p21^WAF1/KIP1^ induction is independent of p53 status in primary human lymphatic endothelial cells. In addition, we demonstrated that p53 knockdown by siRNA does not reverse TSA mediated inhibition of proliferation in lymphatic endothelial cells, whereas p21^WAF1/KIP1^ depletion completely reversed the growth inhibition of lymphatic endothelial cells after TSA treatment. In our study we demonstrated that p27^KIP1^ protein, like p21^WAF1/KIP1^, increased due to TSA-treatment, suggesting that p27^KIP1^ could be also involved in TSA-induced G1-arrest in LECs. The data support recent results showing a possible correlation between G1-arrest and induction of p27^KIP1^ in various cancer cell lines [71]. Interestingly, p27^KIP1^ and p53 mRNA were downregulated after TSA-treatment, whereas protein levels increased at a relatively low dose and at an early time point, which implied that p27^KIP1^ and p53 protein accumulation is possibly regulated at a post-translational level. Our observations have been confirmed by various studies that protein modification of p27^KIP1^ and p53 by phosphorylation or acetylation would affect the stability of modified proteins by modulating ubiquitination dependent proteasome proteolysis [72, 73]. For example, it has been found that HDACi induced apoptosis in human hepatoma HepG2 cells. Apoptosis was accompanied with the presence of acetylated p53 together with acetylated forms of histones and histone acetyltransferases p300 and PCAF, indicating that HDACi influences p53 protein stability [74]. Uehara and colleagues demonstrated that the HDACi vorinostat enhanced protein stability of p27^KIP1^ and p21^WAF1/KIP1^, without concomitant induction of p27^KIP1^ mRNA, through negative regulation of Skp2 and Cks1 in human breast cancer cells [75]. In the study of Chen et al., it has been shown that the HDACi NaB induced p27^KIP1^-dependent G1-Arrest in murine fibroblasts. Interestingly, p27^KIP1^ mRNA-levels of butyrate-treated fibroblasts were decreased; instead, the stability of the p27^KIP1^ protein was found to be increased upon HDACi-treatment [76]. Since HDACi could induce the acetylation of multiple proteins with subsequent control of the ubiquitin-proteasome pathway, this could be one of the additional mechanisms to affect p27^KIP1^ and p53 protein degradation, thus inducing p27^KIP1^ and p53 accumulations in LECs, which need to be confirmed in future studies. Additionally, the exactly function of p27^KIP1^ in TSA-induced growth arrest in LECs warrants further investigation of its role as a promising therapeutic target in controlling lymphangiogenesis in pathological conditions.

## Conclusions

In summary, our study provides strong evidence that TSA – a potent pan-HDACi - mediates distinct anti-lymphangiogenic activities in primary human lymphatic endothelial cells by activating the intrinsic apoptotic pathway and inducing cell cycle arrest. TSA-induced cell death was associated with downregulating the anti-apoptotic proteins cIAP-1/2. Furthermore, we observed that cell cycle arrest is associated with modulation of the important checkpoint control proteins p21^WAF1/KIP1^, p27^KIP1^, p53 and cyclin A resulting in G0/G1-arrest. Induction of p21^WAF1/KIP1^ in our study was associated with increased nuclear accumulation and transcriptional activation of the promoter via Sp1/3 binding sites. We have also demonstrated that growth inhibition of lymphatic endothelial cells after TSA treatment is due to p21^WAF1/KIP1^ induction but independent of p53 expression status. These findings provide strong evidence for the use of HDACi in the treatment of malignancies associated with increased lymphangiogenesis. In conclusion, we identified in our study new targets for TSA mediated anti-lymphangiogenic properties in primary human lymphatic endothelial cells, which may be important for the in vivo constellation.

## Additional files

Additional file 1: Figure S1. Effects of TSA on important cell cycle regulators. Densitometric analysis of Western blots. TSA induced the expression of p21, p27 and p53 in a concentration-dependent manner. LECs that were left untreated (solvent only) or were treated with TSA in a concentration-dependent manner for 24 h as indicated. The western blot bands were quantified by densitometry; absorbencies of p21, p27 and p53 bands were corrected for loading differences based on the corresponding tubulin bands. Experiments were performed at least three times, and the results are shown as mean ± SD. *p < 0.05 vs ctrl., ns = not significant. (JPG 28 kb) Additional file 2: Figure S2. TSA induces apoptosis through activation of the intrinsic pathway. TSA increased the cleavage of the caspases 3, 7 and 9. LECs that were left untreated (solvent only) or were treated with TSA in a concentration-dependent manner for 24 h as indicated. The western blot bands were quantified by densitometry; absorbencies of cleaved caspase 3, 7 and 9 bands were corrected for loading differences based on the corresponding tubulin bands. Experiments were performed at least three times, and the results are shown as mean ± SD. *p < 0.05 vs ctrl., ns = not significant. (JPG 31 kb) Additional file 3: Figure S3. Effects of p21 and p53 depletion in LECs by siRNA with and without TSA-treatment. Densitometric analysis of Western blots. Cells were treated with siRNA against p21 (A), p53 (B) and control siRNA and were exposed to 400 nM TSA or solvent only (=Ethanol; 100 %) for 24 h as indicated. As expected, depletion of p21 and p53 protein could not be restored after TSA treatment. The western blot bands were quantified by densitometry; absorbencies of p21 and p53 bands were corrected for loading differences based on the corresponding tubulin bands. Experiments were performed at least three times, and representative results are shown. (JPG 35 kb) Additional file 4: Figure S4. Depletion of p53 did not reversed TSA-induced upregulation of p21. Densitometric analysis of Western blots. Cells were treated with siRNA against p53 and control siRNA and were exposed to 400 nM TSA or solvent only (=Ethanol; 100 %) for 24 h as indicated. We found, that depletion of p53 did not affected TSA-induced upregulation of p21 protein (Fig. 7b). The western blot bands were quantified by densitometry; absorbencies of p21 bands were corrected for loading differences based on the corresponding tubulin bands. Experiments were performed at least three times, and representative results are shown. (JPG 21 kb)

## Abbreviations

CHX: Cycloheximide
cIAP1 and cIAP2: Cellular inhibitor of apoptosis 1 and 2
FACS: Fluorescenceactivated cell sorting analysis
HDAC: Histon deacetylase
HDACi: Histon deacetylase inhibitors
LEC: Lymphatic endothelial cells
NaB: Sodium butyrate
RT-PCR: Reverse-transcription polymerase chain reaction
SDS-PAGE: Sodium dodecyl sulphate–polyacrylamide gel electrophoresis
siRNA: Small interfering RNA
TSA: Trichostatin A
VPA: Valproic acid
