# Supplementary figures and images for: The histone deacetylase inhibitor trichostatin a decreases lymphangiogenesis by inducing apoptosis and cell cycle arrest via p21-dependent pathways
Source: BMC Cancer. 2016 Sep 30;16:763. doi: 10.1186/s12885-016-2807-y (PMC5045659; doi:10.1186/s12885-016-2807-y)

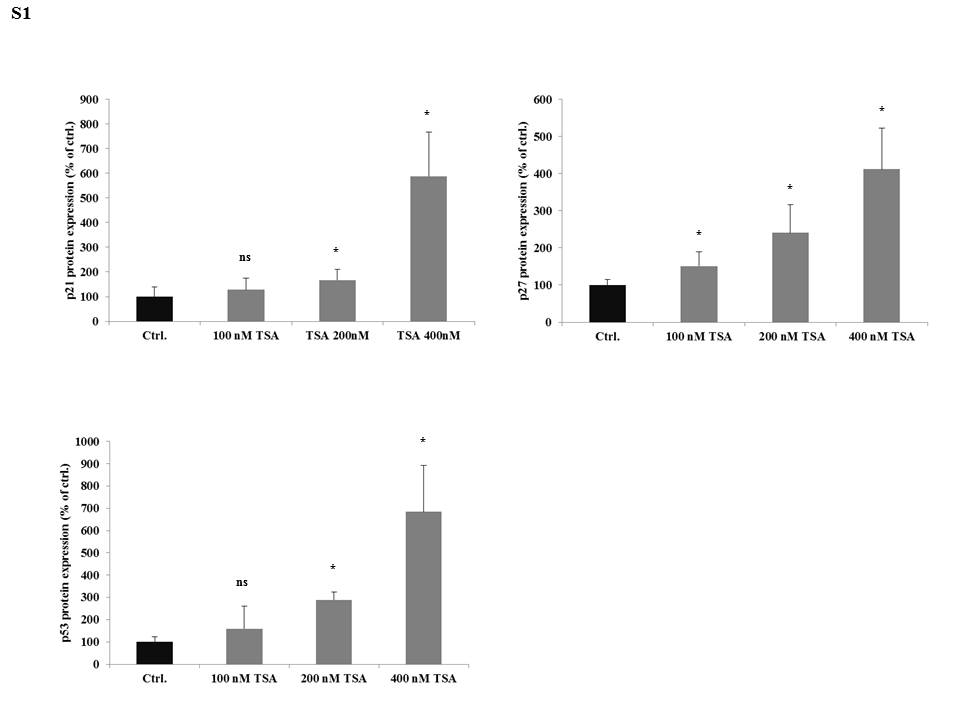

Supplement: Additional file 1: Figure S1. — Effects of TSA on important cell cycle regulators. Densitometric analysis of Western blots. TSA induced the expression of p21, p27 and p53 in a concentration-dependent manner. LECs that were left untreated (solvent only) or were treated with TSA in a concentration-dependent manner for 24 h as indicated. The western blot bands were quantified by densitometry; absorbencies of p21, p27 and p53 bands were corrected for loading differences based on the corresponding tubulin bands. Experiments were performed at least three times, and the results are shown as mean ± SD. *p < 0.05 vs ctrl., ns = not significant. (JPG 28 kb) [file 12885_2016_2807_MOESM1_ESM.jpg]

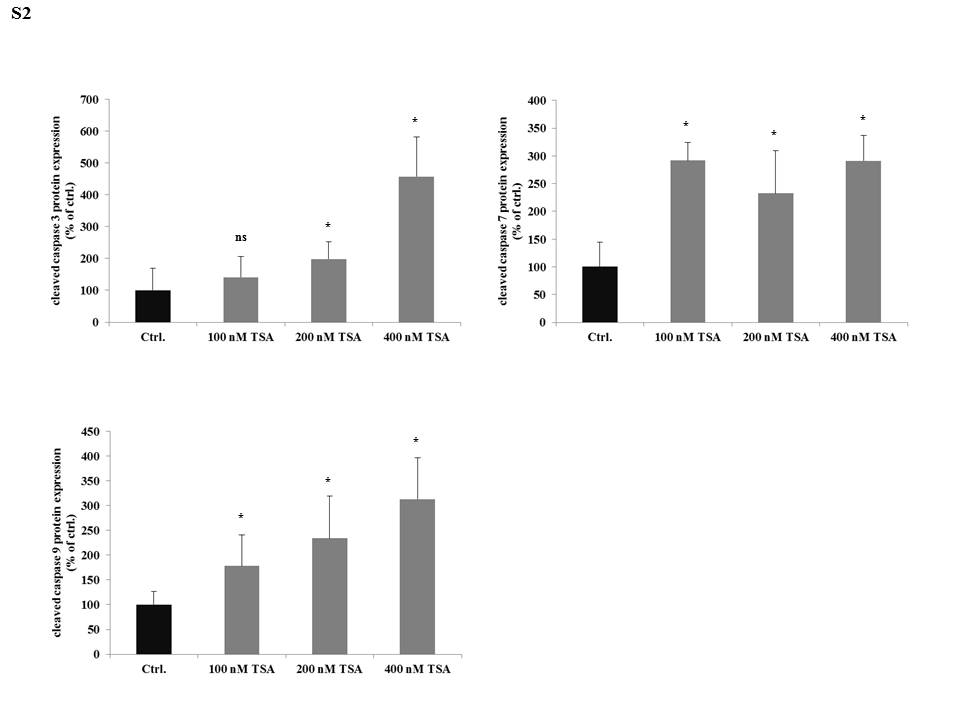

Supplement: Additional file 2: Figure S2. — TSA induces apoptosis through activation of the intrinsic pathway. TSA increased the cleavage of the caspases 3, 7 and 9. LECs that were left untreated (solvent only) or were treated with TSA in a concentration-dependent manner for 24 h as indicated. The western blot bands were quantified by densitometry; absorbencies of cleaved caspase 3, 7 and 9 bands were corrected for loading differences based on the corresponding tubulin bands. Experiments were performed at least three times, and the results are shown as mean ± SD. *p < 0.05 vs ctrl., ns = not significant. (JPG 31 kb) [file 12885_2016_2807_MOESM2_ESM.jpg]

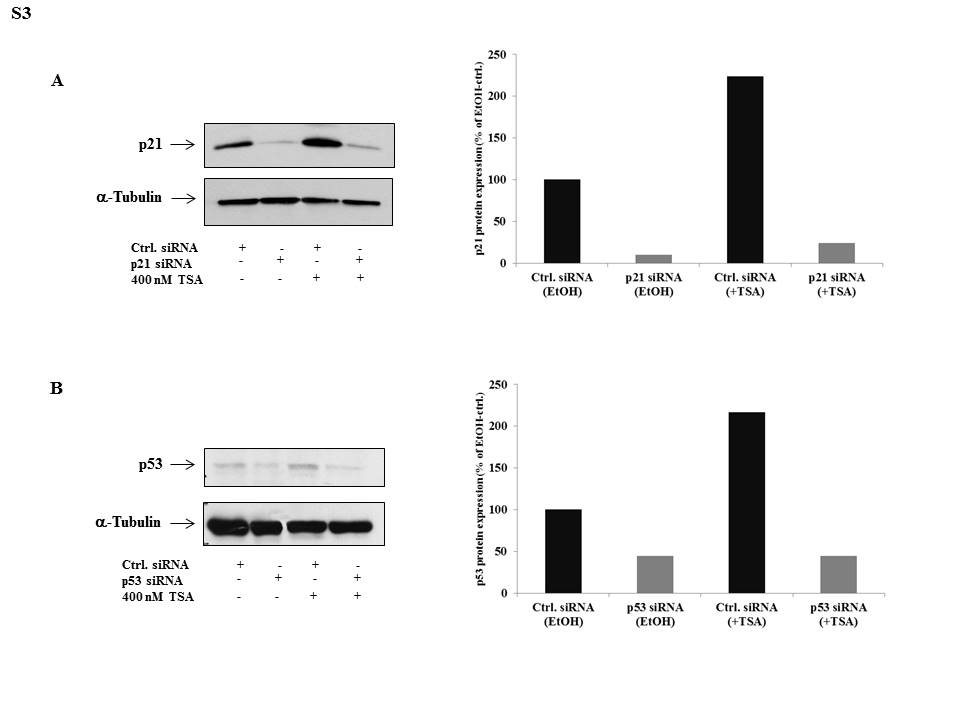

Supplement: Additional file 3: Figure S3. — Effects of p21 and p53 depletion in LECs by siRNA with and without TSA-treatment. Densitometric analysis of Western blots. Cells were treated with siRNA against p21 (A), p53 (B) and control siRNA and were exposed to 400 nM TSA or solvent only (=Ethanol; 100 %) for 24 h as indicated. As expected, depletion of p21 and p53 protein could not be restored after TSA treatment. The western blot bands were quantified by densitometry; absorbencies of p21 and p53 bands were corrected for loading differences based on the corresponding tubulin bands. Experiments were performed at least three times, and representative results are shown. (JPG 35 kb) [file 12885_2016_2807_MOESM3_ESM.jpg]

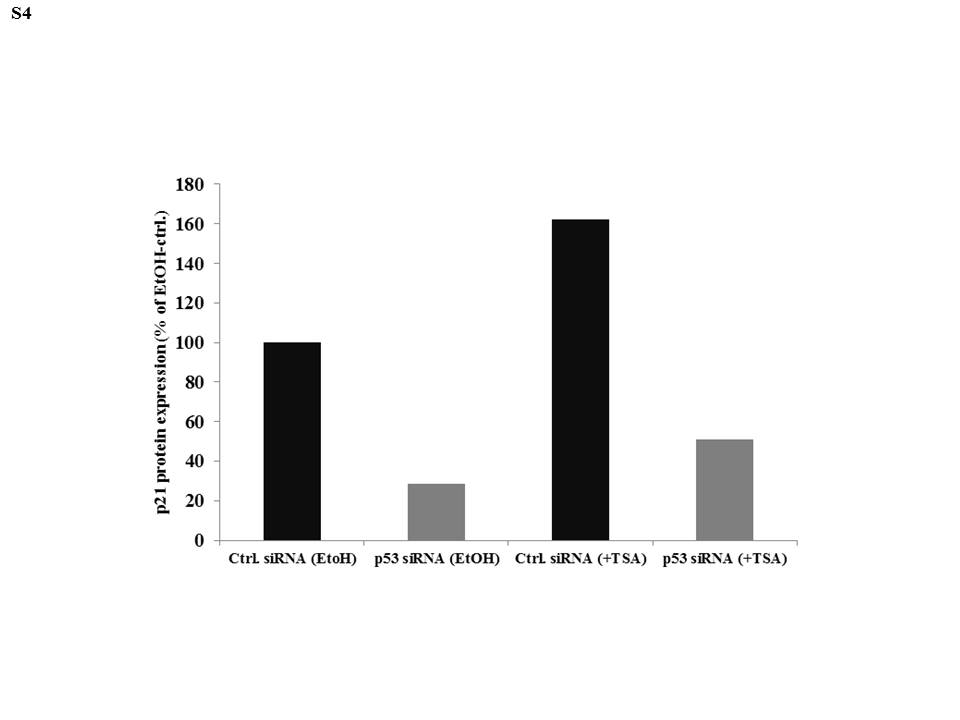

Supplement: Additional file 4: Figure S4. — Depletion of p53 did not reversed TSA-induced upregulation of p21. Densitometric analysis of Western blots. Cells were treated with siRNA against p53 and control siRNA and were exposed to 400 nM TSA or solvent only (=Ethanol; 100 %) for 24 h as indicated. We found, that depletion of p53 did not affected TSA-induced upregulation of p21 protein (Fig. 7b). The western blot bands were quantified by densitometry; absorbencies of p21 bands were corrected for loading differences based on the corresponding tubulin bands. Experiments were performed at least three times, and representative results are shown. (JPG 21 kb) [file 12885_2016_2807_MOESM4_ESM.jpg]
